# LncRNA SH3BP5-AS1 promotes hepatocellular carcinoma progression by sponging miR-6838-5p and activation of PTPN4

**DOI:** 10.18632/aging.205811

**Published:** 2024-05-16

**Authors:** Xinyang Zhao, Xinfeng Zhu, Chaowen Xiao, Zouxiao Hu

**Affiliations:** 1 Department of Hepatobiliary Surgery, The Central Hospital of Wuhan, Tongji Medical College, Huazhong University of Science and Technology, Wuhan 430014, Hubei, China

**Keywords:** SH3BP5-AS1, hepatocellular carcinoma, miR-6838-5p, PTPN4, HIF-1α

## Abstract

Background: Long noncoding RNAs (LncRNAs) have been demonstrated to have significant roles in the carcinogenesis of hepatocellular carcinoma (HCC). In this work, we sought to determine LncRNA SH3BP5-AS1’s function and mechanism in the emergence of HCC.

Results: First, we discovered that the advanced tumor stage was strongly correlated with high levels of LncRNA SH3BP5-AS1 expression in HCC. MiR-6838-5p expression was down-regulated and inversely correlated with SH3BP5-AS1 expression. Additionally, overexpression of SH3BP5-AS1 boosted cell invasion, migration, and proliferation. The oncogenic effects of the inhibitor of miR-6838-5p were eliminated when PTPN4 was suppressed, following the identification of PTPN4 as a direct target of miR-6838-5p. In addition, SH3BP5-AS1 promoted cellular glycolysis via miR-6838-5p sponging and PTPN4 activation. Lastly, by directly interacting to the promoter of SH3BP5-AS1, HIF-1α could control the transcription of the gene.

Conclusions: Our research suggests that SH3BP5-AS1 controls miR-6838-5p/PTPN4 in order to act as a new carcinogenic LncRNA during the growth of HCC cells.

Methods: The expression levels of SH3BP5-AS1, miR-6838-5p and PTPN4 were detected by qRT-PCR and Western blot. The effects of LncRNA SH3BP5-AS1/miR-6838-5p/PTPN4 on the proliferation, metastasis and glycolysis of HCC cells were clarified by experimental cellular functionality assays, cell derived xenograft and Glycolysis assay.

## INTRODUCTION

Hepatocellular carcinoma (HCC), which is one of the most common malignancies, has a significant morbidity and mortality rate that is increasing at a steady rate [1]. Despite the fact that systemic therapy for HCC has advanced, strategies to achieve successful treatment are limited due to poor prognosis and high recurrence rates [2, 3]. Furthermore, HCC still presents a serious health risk to individuals and has an appalling prognosis for patients [4]. Thus, in an effort to improve the prevention and early detection of HCC, in addition to patient survival, there is a need to detect its potential molecular mechanisms, so that overall patient prognosis and effective individualized treatment can be established.

Noncoding RNA with transcripts longer than 200 nucleotides is known as long noncoding RNA (LncRNA). LncRNAs play major roles in epigenetic control, post-transcriptional regulation and gene transcription in biology [5]. To date, a variety of LncRNAs have been found to be abnormally expressed in HCC, and they have also been demonstrated to be important for HCC cell invasion, proliferation, metastasis, apoptosis, and chemosensitivity [6]. For example, Li et al. found that LncRNA SNHG5 controls the Wnt and UPF1 signaling pathways to enhance HCC growth and tumor stem cell-like characteristics [7]. Zhang et al. reported that LncRNA HEPFAL accelerates iron death in HCC through the regulation of SLC7A11 ubiquitination [8]. Feng et al. demonstrate that LncRNA PCNAP1 regulates hepatitis B virus replication and promotes HCC tumor growth [9]. According to Wang et al., in hypoxic environments, exosomal LncRNA HMMR-AS1 regulates macrophage polarization and influences HCC development via the miR-147a/ARID3A axis [10]. The pathophysiology of HCC is better understood thanks to the study’s findings, which may potentially result in the creation of a unique and trustworthy molecular marker for the early identification of the disease, the estimation of the likelihood that it will spread, and a new target for chemotherapy and radiation treatment.

In some previous studies, researchers have found that SH3BP5-AS1 may be involved in the metastasis and aging process of tumor cells, thus affecting the clinical prognosis of lung adenocarcinoma patients [11, 12]. There are also studies that show that SH3BP5-AS1 activated Wnt signaling pathway by sponging miR-139-5p, upregulating CTBP1 expression, and contributing to the sensitivity of pancreatic cancer cells to gemcitabine [13]. However, its role and mechanism in HCC need to be further explored.

In the present study, we showed that the tissues and cells of HCC expressed a significant amount of the LncRNA SH3BP5-AS1. By controlling the miR-6838-5p/PTPN4 axis and encouraging HCC cell glycolysis, LncRNA SH3BP5-AS1 accelerated the development of HCC. In conclusion, the results of our study have the potential to aid in the detection and management of HCC.

## RESULTS

### LncRNA SH3BP5-AS1 was over-expressed in HCC cells and tissues

**Figure 1:**
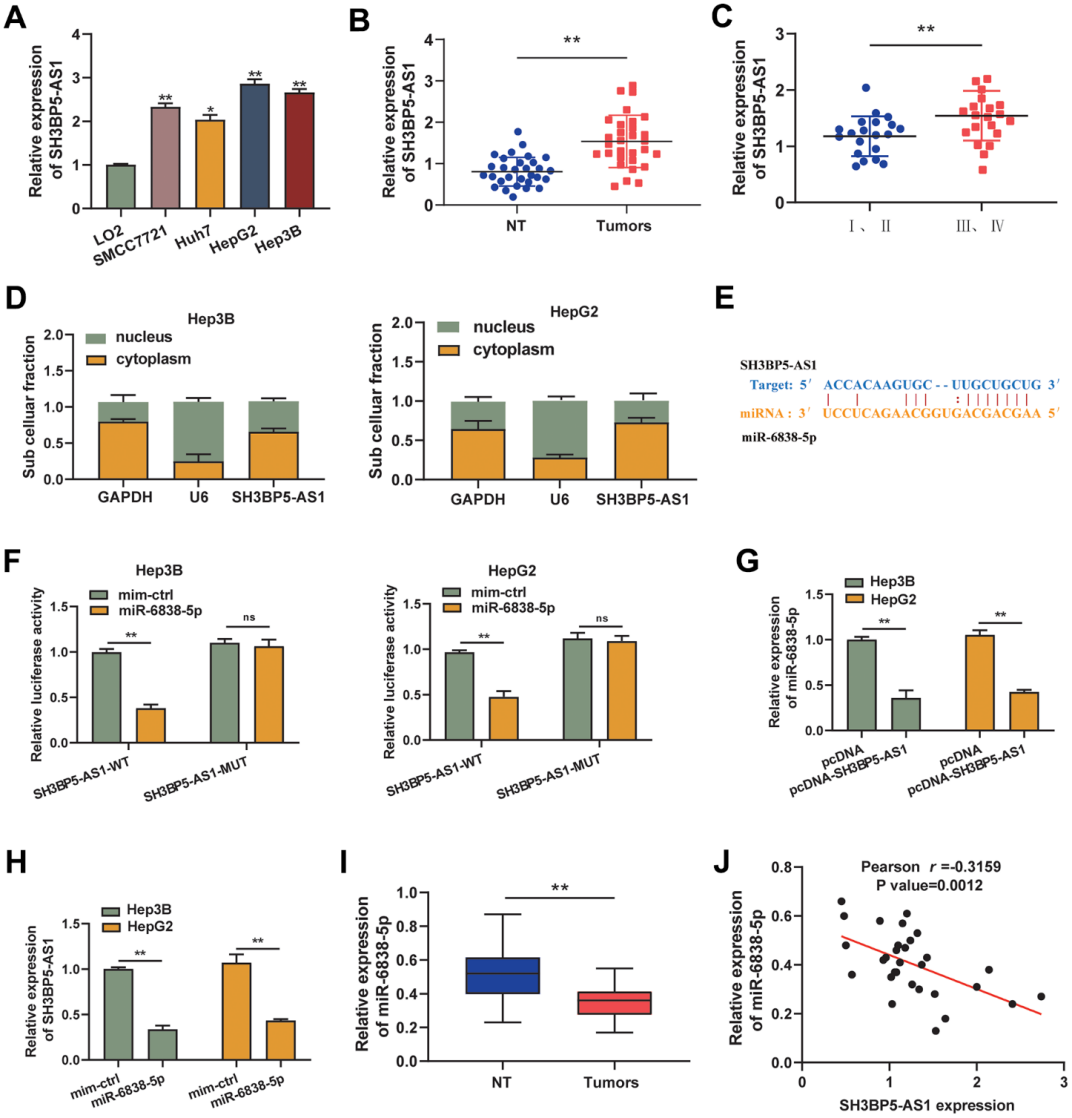
**SH3BP5-AS1 and miR-6838-5p have a relationship in HCC.** (**A**, **B**) HCC cells and tissues showed increased SH3BP5-AS1 expression. (**C**) The patients’ advanced tumor stage was indicated by the elevated SH3BP5-AS1. (**D**) The position of SH3BP5-AS1 was identified using subcellular fractionation analysis. (**E**) The binding sites of SH3BP5-AS1 and miR-6838-5p. (**F**) MiR-6838-5p inhibited the activity of the luciferase gene in SH3BP5-AS1 wild-type (SH3BP5-AS1-WT), but not in SH3BP5-AS1 mutant (SH3BP5-AS1-MUT). (**G**) SH3BP5-AS1 prevented MiR-6838-5p from being expressed. (**H**) MiR-6838-5p mimics decreased SH3BP5-AS1 expression. (**I**) In HCC tissues, miR-6838-5p expression was markedly downregulated. (**J**) A negative correlation was observed between SH3BP5-AS1 and miR-6838-5p expression.

First, we looked for the expression of the LncRNA SH3BP5-AS1 in HCC cell lines (SMCC7721, Huh7, HepG2 and Hep3B) as well as in human hepatic epithelial cell lines (LO2). In HCC cell lines, we discovered that LncRNA SH3BP5-AS1 expression was elevated (Figure 1A). In keeping with this, we also demonstrated that HCC tissue had highly elevated LncRNA SH3BP5-AS1 expression (Figure 1B). Furthermore, in tissues with HCC, there was a substantial correlation between SH3BP5-AS1 expression and TNM stage (Figure 1C). LncRNA SH3BP5-AS1 was upregulated overall in HCC.

### The regulatory relationship between LncRNA SH3BP5-AS1 and miR-6838-5p

In this study, we used the distribution of LncRNAs in cellular components as the basis for LncRNA regulatory mechanisms. In agreement with this, we identified the distribution of SH3BP5-AS1 in HCC cells, as well as demonstrated that SH3BP5-AS1 was primarily concentrated in the cytoplasm (Figure 1D). In view of the observations that SH3BP5-AS1 has a strong ability to interact with cytoplasmic miRNAs. For our search, we used the Starbase v3.0 database to look for possible miRNAs that SH3BP5-AS1 might be able to control. We demonstrated that SH3BP5-AS1 was capable of binding to miR-6838-5p (Figure 1E). In a luciferase activity experiment, it was found that co-transfection of miR-6838-5p and SH3BP5-AS1 wild type led to a dramatic reduction in luciferase activity, while mutation of SH3BP5-AS1 lost this function (Figure 1F). Overexpression of SH3BP5-AS1 resulted in the downregulation of miR-6838-5p expression (Figure 1G). Similarly, miR-6838-5p has been shown to mimic a significant reduction of SH3BP5-AS1 expression in HCC cells (Figure 1H). Moreover, we discovered that HCC tissues have downregulated miR-6838-5p (Figure 1I). Moreover, it was shown that there was a negative correlation between the expression of miR-6838-5p and SH3BP5-AS1 in HCC tissues (Figure 1J). The aforementioned information showed that in HCC, SH3BP5-AS1 and miR-6838-5p had a negative connection.

### HCC cells’ proliferation, migration, and invasion were controlled by the LncRNA SH3BP5-AS1/miR-6838-5p axis

**Figure 2:**
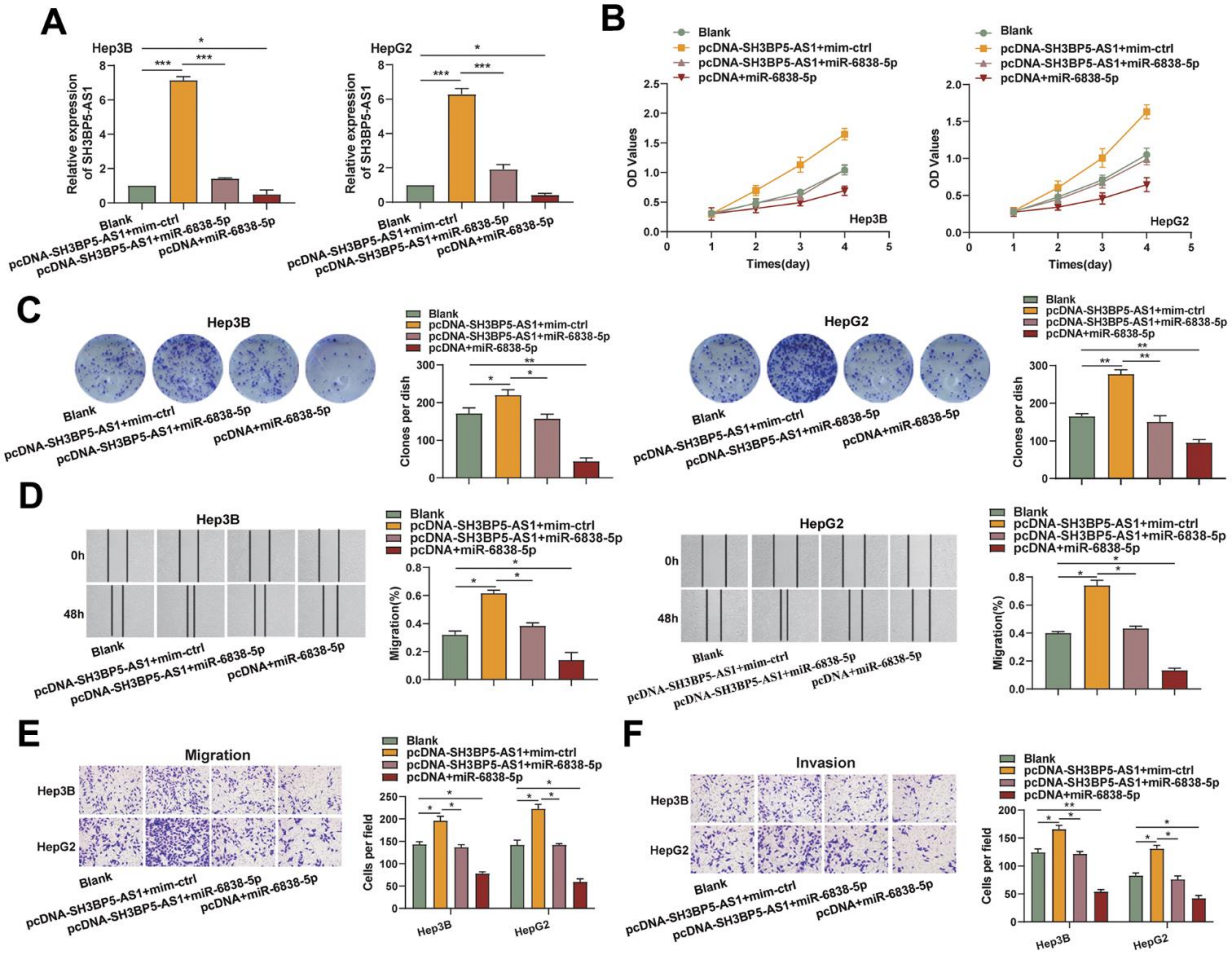
**The impact of SH3BP5-AS1 was diminished by miR-6838-5p.** (**A**) SH3BP5-AS1 expression in HCC cells transfected with pcDNA-SH3BP5-AS1 and miR-6838-5p (or mim-ctrl) was determined by qRT-PCR assay. (**B**, **C**) CCK-8 and colony formation assays were used to investigate the effect of the SH3BP5-AS1/miR-6838-5p axis on the proliferation capacity of the HCC cells. (**D**–**F**) Wound healing and transwell assays were used to assess the impact of the SH3BP5-AS1/miR-6838-5p axis on the migration and invasion capacity of the HCC cells.

Additionally, we discovered that the SH3BP5-AS1/miR-6838-5p axis functions biologically in HCC cells. In the beginning, we created HCC cells that overexpressed the LncRNA SH3BP5-AS1, miR-6838-5p, and both of these RNAs together (Figure 2A). The CCK-8 and colony formation assays revealed that the ectogenous SH3BP5-AS1-induced miR-6838-5p greatly reduced the quantity of cell proliferation (Figure 2B, 2C). In the cell scratch assay, SH3BP5-AS1 was shown to promote migratory ability of the HCC cells, whereas miR-6838-5p was able to partially abolish the effect (Figure 2D). Furthermore, overexpression of SH3BP5-AS1 was shown to increase the number of cells that invaded and migrated per field; on the other hand, overexpression of miR-6838-5p counteracted the effect of SH3BP5-AS1 on HCC cell invasion and migration (Figure 2E, 2F). According to these findings, the LncRNA SH3BP5-AS1/miR-6838-5p axis regulated the HCC cells’ capacity to proliferate, migrate, and invade.

### PTPN4 was a functional target of miR-6838-5p

**Figure 3:**
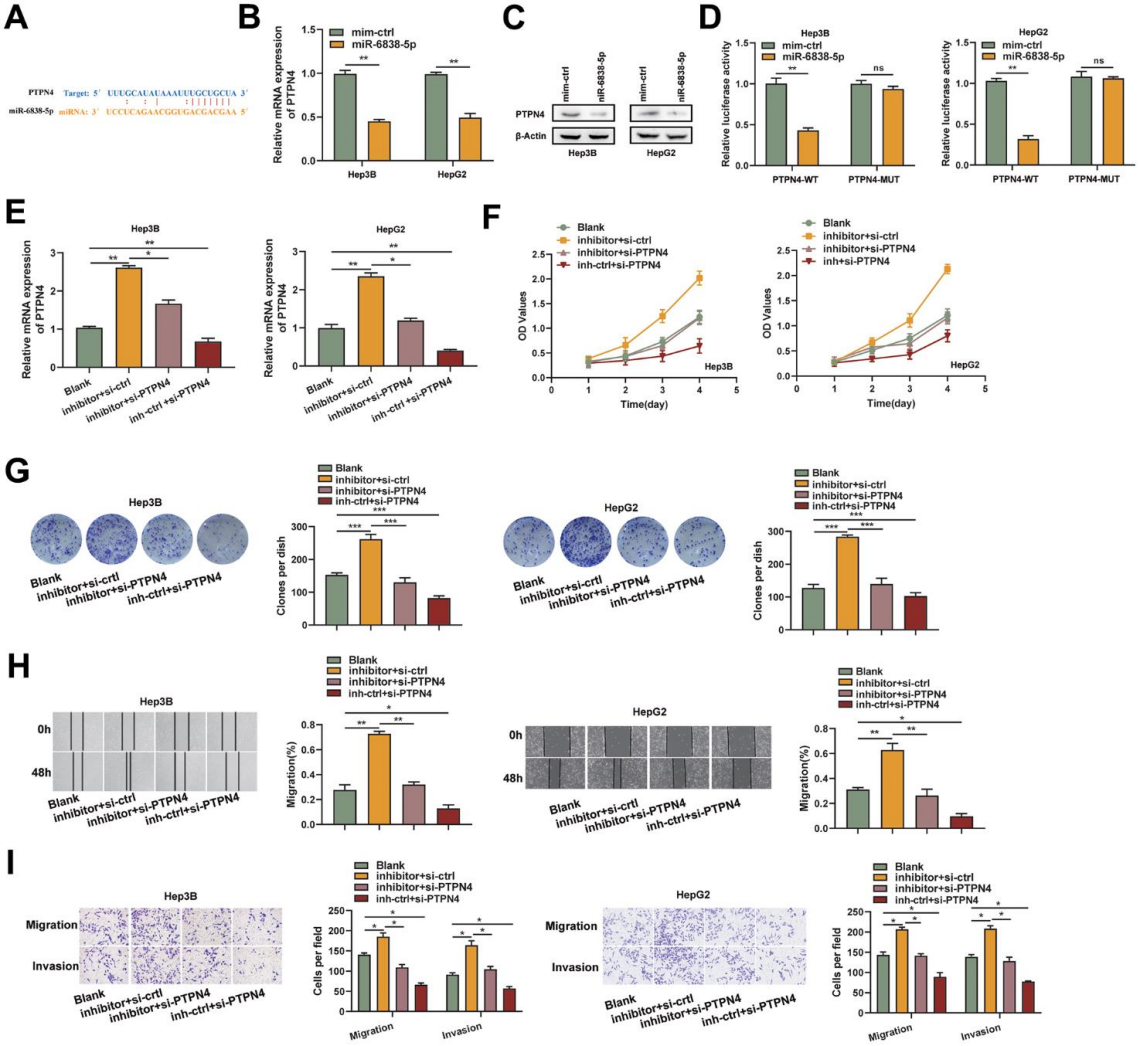
**PTPN4 was a functional target of miR-6838-5p.** (**A**) The PTPN4 mRNA 3-UTR’s miR-6838-5p binding location. (**B**, **C**) MiR-6838-5p prevented PTPN4’s mRNA and protein from being expressed. (**D**) Luciferase activity was significantly decreased when PTPN4-WT and miR-6838-5p were co-transfected. (**E**) The qRT-PCR technique was used to measure the mRNA level of PTPN4 in the HCC cells. (**F**, **G**) PTPN4 silencing prevented the miR-6838-5p inhibitor’s promotion effect on HCC cells’ growth. (**H**, **I**) The cell scratch and transwell assays were used to identify the migration and invasion capacity of HCC cells.

The potential functional target genes that SH3BP5-AS1 is known to control were estimated using three online tools: TargetScan, miRDB, and microRNA [14]. We found that miR-6838-5p may have PTPN4 as a possible target (Figure 3A). After the HCC cells were treated with the miR-6838-5p mimic, there was a considerable drop in the levels of PTPN4’s mRNA and protein (Figure 3B, 3C). MiR-6838-5p mimics dramatically decreased the luciferase activity of PTPN4-WT cells, but had no effect on PTPN4-MUT cells, according to luciferase activity assays (Figure 3D). We used an inhibitor of miR-6838-5p and a small interfering RNA of PTPN4 to treat HCC cells and found that inhibiting miR-6838-5p led to an increase in PTPN4 mRNA and that inhibiting PTPN4 could reverse this effect (Figure 3E). CCK-8 and colony formation experiments demonstrated that inhibiting miR-6838-5p increased HCC cells’ proliferation ability whereas PTPN4 silencing had the opposite effect (Figure 3F, 3G). HCC cells were encouraged to migrate and invade when MiR-6838-5p was inhibited; however, this impact was somewhat counteracted by PTPN4 knockdown (Figure 3H, 3I). These results suggested that PTPN4 was a functional target of miR-6838-5p.

### SH3BP5-AS1/miR-6838-5p/PTPN4 axis regulated glycolysis in HCC

**Figure 4:**
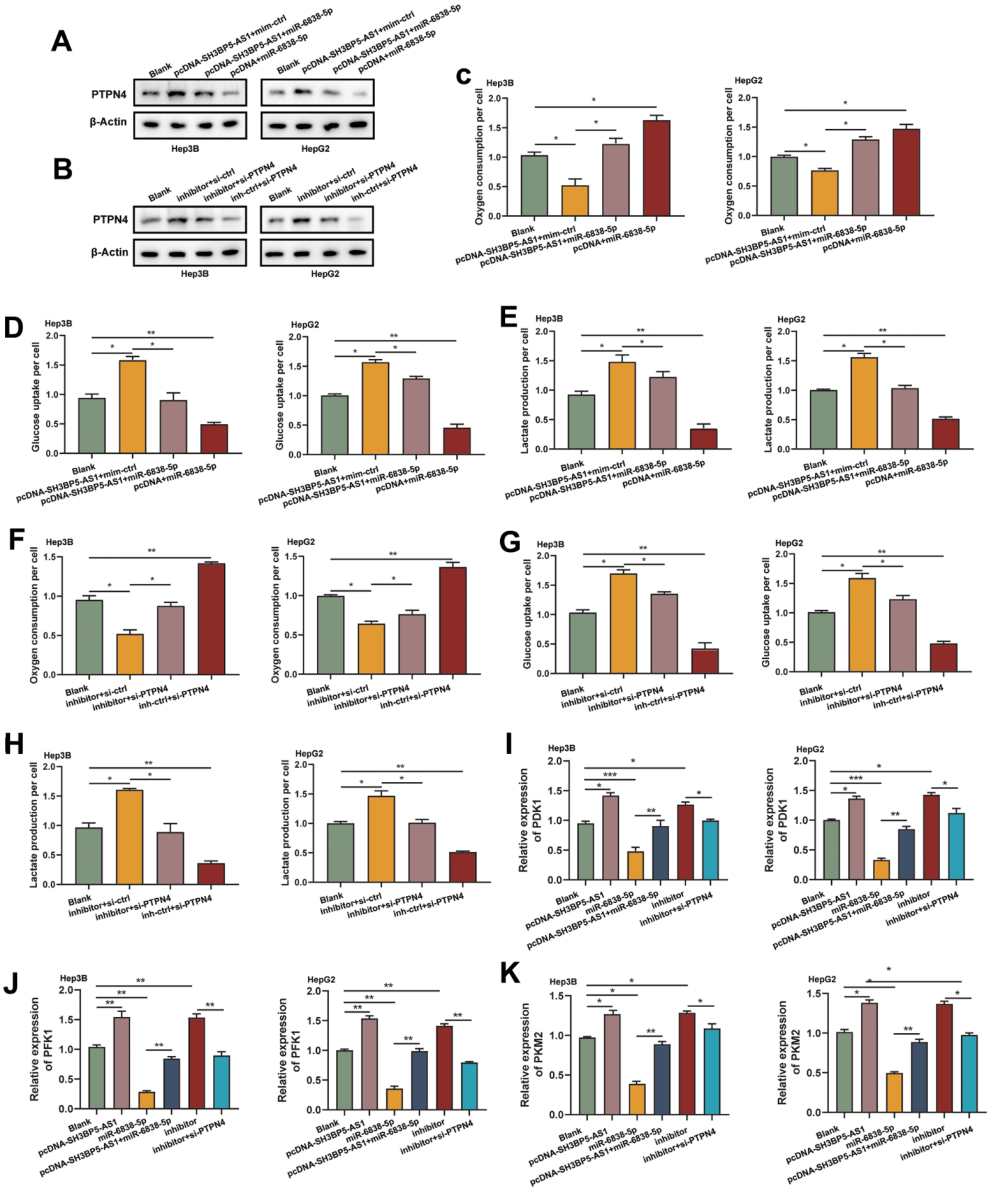
**The SH3BP5-AS1/miR-6838-5p/PTPN4 axis regulated glycolysis in HCC.** (**A**, **B**) By sponging miR-6838-5p, SH3BP5-AS1 activated PTPN4. (**C**–**E**) Ectogenic miR-6838-5p lessened the effect of SH3BP5-AS1 on glucose absorption and lactate production. (**F**–**H**) Knockdown of PTPN4 reversed the increasement in HCC cells’ glycolysis brought on by the suppression of miR-6838-5p. (**I**–**K**) Using qRT-PCR experiment, the expression levels of PDK1, PFK1, and PKM2 in HCC cells were investigated.

The objective of our study was to learn more about how the SH3BP5-AS1/miR-6838-5p/PTPN4 axis affects HCC cells glycolysis. We first demonstrated that overexpression of SH3BP5-AS1 upregulated the level of PTPN4, consistent with the action of miR-6838-5p inhibitor. The ectogenous SH3BP5-AS1 and miR-6838-5p inhibitor’s effect on PTPN4 was reversed by miR-6838-5p or knockdown of PTPN4 (Figure 4A, 4B). These findings demonstrated that SH3BP5-AS1 acts as a ceRNA for miR-6838-5p, which modulates PTPN4 expression in HCC. Later, we discovered that SH3BP5-AS1 could enhance the level of glucose metabolism and lactate production in HCC. However, miR-6838-5p could block the effect of SH3BP5-AS1 on HCC glucose metabolism and lactate production (Figure 4C–4E). Furthermore, we discovered that silencing PTPN4 might undo the miR-6838-5p inhibitor’s effects on glucose metabolism and lactate generation levels (Figure 4F–4H). Furthermore, overexpression of SH3BP5-AS1 and treatment with the inhibitor of miR-6838-5p in HCC cells boosted the mRNA level of three key enzymes of glycolysis, including PDK1, PFK1 and PKM2. While the HCC cells treated with miR-6838-5p mimics or PTPN4 siRNA decreased their expression level (Figure 4I–4K). Our data displayed that the SH3BP5-AS1/miR-6838-5p/PTPN4 axis could regulate glycolysis in HCC.

### SH3BP5-AS1 accelerated HCC cell proliferation *in vivo*

**Figure 5:**
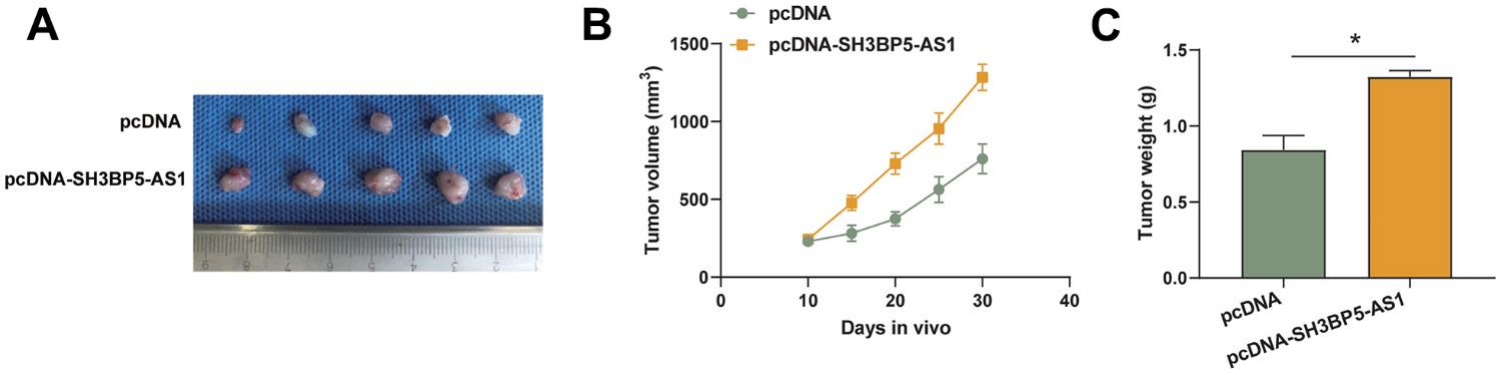
**SH3BP5-AS1 accelerated HCC cell proliferation *in vivo*.** (**A**–**C**) Tumor xenograft assay in the indicated HCC cells.

We generated the stably overexpressing SH3BP5-AS1 HCC cell line and the matching negative control cell line and carried out an animal experiment to ascertain how SH3BP5-AS1 influences HCC cell growth *in vivo*. According to the findings, SH3BP5-AS1 facilitated the development of HCC cells *in vivo* (Figure 5A–5C).

### HIF-1α regulated SH3BP5-AS1 transcription by directly binding to its promoter

**Figure 6:**
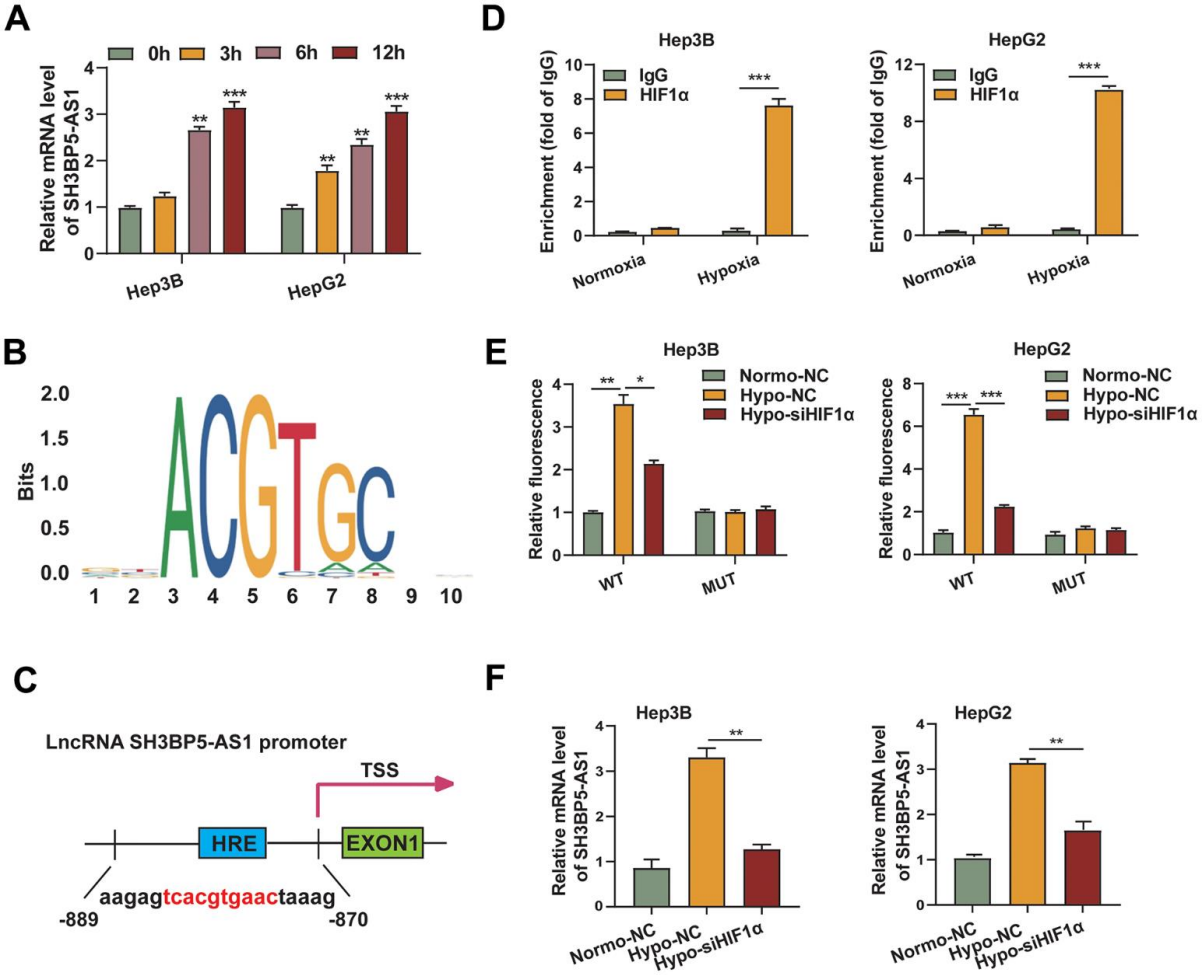
**HIF-1α regulated SH3BP5-AS1 transcription by directly binding to its promoter.** (**A**) The mRNA level of SH3BP5-AS1 in the hypoxic HCC cells was measured using a qRT-PCR experiment. (**B**) HIF-1α binding motif. (**C**) Possible HIF-1α binding location in the SH3BP5-AS1 promoter. (**D**) CHIP-qPCR assay. (**E**) Dual luciferase reporter assay in the HCC cells. (**F**) qRT-PCR experiment in the HCC cells.

Interestingly, following culture under hypoxic circumstances, the amount of SH3BP5-AS1 mRNA was considerably elevated in HCC cells (Figure 6A). As a result, we tended to look into whether hypoxia-inducible factors regulated SH3BP5-AS1. A binding region (-884 to -875) in the SH3BP5-AS1 promoter was predicted to be bound with HIF-1α by comparing the sequence of the promoter to the HIF-1α motif (Figure 6B, 6C). An qRT-PCR assay was conducted to provide additional confirmation, and the findings showed that the CHIP products obtained from an anti-HIF-1α antibody significantly increased the HIF-1α binding motif in the SH3BP5-AS1 promoter. Furthermore, it was more noticeable in hypoxic environments (Figure 6D). Moreover, HIF-1α silencing diminished the effect of hypoxia, and hypoxia increased the fluorescence intensity in HCC cells transfected with the wild type (WT) binding site sequence, according to the results of the dual-luciferase reporter test. However, this impact was not observed in the groups transfected with the mutated (MUT) binding site sequence (Figure 6E). Moreover, SH3BP5-AS1 mRNA level was enhanced under hypoxia condition, and silencing of HIF-1α could partly reverse the expression of SH3BP5-AS1 (Figure 6F). All above implied that HIF-1α may regulate the transcription of SH3BP5-AS1 via binding with its promoter.

## DISCUSSION

A previous study had shown that LncRNAs play key roles in HCC development. In our investigation, it was discovered that the LncRNA SH3BP5-AS1 was substantially expressed in the tissues and cells of HCC. Parallel to this, we discovered that LncRNA SH3BP5-AS1 overexpression encourages HCC cells growth, migration and invasion. It has been demonstrated that LncRNA SH3BP5-AS1 has an oncogenic function in HCC.

Numerous studies have discovered that LncRNAs may behave as molecular sponges or ceRNAs to control miRNA and biological activity. Chen et al. showed that LncTUG1 might speed up the development of HCC by acting on the mTOR/S6K pathway and the miR-144-3p/RRAGD axis [15]. Gu et al. showed that lncRNA HOTAIRM1 overexpression promotes levatinib resistance through the downregulation of miR-34a and activation of autophagy in HCC [16]. By controlling miR-708-5p-mediated glycolysis, Zhang et al. hypothesized that LncRNA MBNL1-AS1 knockdown improved the susceptibility of HCC to ryanodine [17]. In order to prevent Caspase-9 inactivation, Wu et al. revealed that long-chain non-coding RNA cytoskeleton regulator RNA controls the X-1 axis of miR-125a-5p/HS1-related protein in HCC cells [18]. Li et al. discovered that silencing lncRNA HCG18 prevents sorafenib resistance in HCC by regulating GPX4-inhibited iron death by adsorbing miR-450b-5p [19]. In our research, we discovered that LncRNA SH3BP5-AS1 bound to miR-6838-5p and controlled its production and activity. MiR-6838-5p was down-regulated in a variety of cancer types, including breast cancer [20], stomach cancer [21] and Osteosarcoma [22], as demonstrated in the prior work. It has been discovered that miR-6838-5p inhibits the self-renewal and metastasis of human HCC stem cells via deactivating ERK signaling and downregulating CBX4 expression [23]. In line with other studies, we discovered miR-6838-5p repressed the expression of PTPN4 and reduced HCC growth, migration and invasion.

When a tumor develops, malignant cells reorganize their metabolism to meet the biosynthetic demands required to grow their biomass and circumvent the limitations of their microenvironment. One such adaptive process is the persistent stimulation of aerobic glycolysis, which is often referred to as the Warburg effect [24, 25]. Numerous studies have demonstrated the crucial functions that the LncRNA/miRNA axis plays in the Warburg effect by controlling related important enzymes including HK2 and PKM2 [26]. In osteosarcoma, the long noncoding RNA PVT1 has the power to regulate the miR-497/HK2 axis, boosting glycolysis and tumor growth [27]. LncRNA XLOC_006390 acts as a ceRNA against miR-331-3p and positively controls PKM2 expression to aid in the growth and spread of cervical cancer [28]. IGF2BP2 promotes glycolysis mediated by the stability of the lncRNA DANCR and affects the progression of FLT3-ITD + acute myelogenous leukemia [29]. The XIST/miR-93/HIF-1a pathway, which is activated by artemisinin, induces the downregulation of HIF-1a, which limits aerobic glycolysis in thyroid cancer cells [30]. DIO3OS, a long non-coding RNA, is known to promote metabolic reprogramming that promotes glycolysis, which in turn increases breast cancer resistance to aromatase inhibitors [31]. In our current study, we showed that LncRNA SH3BP5-AS1/miR-6838-5p/PTPN4 ameliorated the glycolysis of HCC cells.

Considering all of our findings, we concluded that LncRNA SH3BP5-AS1 acts as a ceRNA by sponging miR-6838-5p to stimulate glycolysis in HCC cells and to promote the expression of PTPN4, which in turn increases the growth, migration and invasion of HCC cells. And HIF-1α could regulate SH3BP5-AS1 transcription by directly binding to its promoter. The findings of our study offer fresh understanding into the mechanism underlying the occurrence of HCC and may provide effective strategy for HCC treatment.

## MATERIALS AND METHODS

### Clinical specimens

We acquired our HCC and the corresponding adjacent non-tumor tissue from patients who were seen at the Wuhan Central Hospital between January 2014 and January 2019. Every patient provided informed permission for the use of the sample, which was approved by the hospital and executed in compliance with the values outlined in the Declaration of Helsinki.

### Cell lines and cell culture

Human HCC cell lines (SMCC7721, Huh7, HepG2, and Hep3B) and normal hepatic epithelial cell LO2 cell lines were obtained from the American Type Culture Collection (Manassas, VA, USA). 10% fetal bovine serum (FBS, Gibco) was added to DMEM (Gibco, Carlsbad, CA, USA) medium used to cultivate the cell lines LO2, SMCC7721, Huh7, HepG2, and Hep3B. Every cell was kept at 37.0° C in a humidified incubator with 5% CO_2_.

### RNA extraction and quantitative real-time PCR (qRT-PCR)

As directed by the manufacturer, all of the RNA was extracted using the TRIzol reagent (Invitrogen, Waltham, MA, USA). RNA was reverse-transcribed into cDNA using Invitrogen’s PrimeScript RT reagent Kit. The ABI 7500 PCR System (ABI) was utilized to conduct qRT-PCR utilizing SYBR Premix Ex Taq II (Takara, Japan). The information of primers used in this study was listed in Supplementary Table 1.

### Western blot analysis

Protease inhibitor, RIPA lysis buffer, and PMSF (Boster, Wuhan, China) were used to extract the proteins. The mixture was centrifuged at 125000 rpm for 15 minutes at 4° C to collect the supernatants. The BCA technique was used to determine the protein concentration. Proteins were separated and added on nitrocellulose members using 10% SDS-PAGE. Membranes were first blocked using 5% nonfat milk, and then the primary antibody PTPN4 (1:1000, Proteintech, Wuhan, China) was incubated at 4° C for a whole night. After that, the relevant secondary antibodies (Boster, Wuhan, China) were incubated for an additional two hours at room temperature. The determination of protein bands was made possible by the chemiluminescence reagent.

### Cell proliferation assay

3×10^3^ cells were plated in triplicate in each well of 96 well plates. Following detection at 24, 48, 72, and 96 hours, a CCK-8 solution (10 μL/well) (Boster, Wuhan, China) was added to the cells in compliance with the manufacturer’s instructions. A multiwell plate reader made by Molecular Devices (San Jose, CA, USA) was used to measure the absorbance at 450 nm after the cells were treated for an additional two hours at 37° C.

### Colony formation assay

Colony formation tests were used to evaluate a cell’s capacity to form colonies. A 6-well plate was plated with 500 cells. After two weeks in culture, the cells were fixed with 4% paraformaldehyde and stained with 0.1% crystal violet solution. Under a light microscope, the colonies were counted once they had more than 50 cells.

### Wound healing assay

Six-well plates were seeded with 1×10^6^ cells per well. The cells were then grown to confluence using a 200 μL micropipette tip to make a scratch wound on the cells, following a PBS wash to remove any remaining cell debris. Progression of cells migration were recorded at 0, 24, 48, and 72 h after scratching with a phase-contrast microscope and digitally photographed (National Research Institute, Health Specialist, Bethesda, MD, USA).

### Cell migration and invasion assays

Tests for cell invasion and migration were performed in transwell chambers. For invasion assays, 5x10^4^ cells were layered at the bottom of transwell plates covered with Matrigel, and 200 μL of serum-free media was added. 10% 700 μL media containing FBS was also introduced into the bottom chamber. After incubation for twenty-four hours, non-invading cells on the top surface membrane were removed. After the cells were dried, dyed with 0.1% crystal violet, and fixed with 4% paraformaldehyde, their vitality was evaluated. For the migration assays, 200 μL of serum-free media without Matrigel was used to seed cells in the top chamber, and 700 μL of 10% FBS medium was applied to the lower chambers. Cells that had migrated into the bottom chamber after 16 hours were fixed in 4% paraformaldehyde, stained with 0.1% crystal violet, dried, and seen under a light microscope.

### Glycolysis assay

The glucose concentration and the lactic acid yield were measured by taking the culture medium supernatant and the cell supernatant, respectively. The amount of extracellular lactic acid was measured using a lactate assay kit (BioVision, Milpitas, CA, USA) in accordance with the manufacturer’s instructions, and the amount of glucose was measured using a glucose test kit (Sigma-Aldrich, St. Louis, MO, USA).

### Animal study

Six-week-old female Balb/c mice were used for all tests, which were conducted in the nude. Balb/c nude mice were given a subcutaneous injection of 2×10^6^ transfected cells into their right armpit. Each mouse’s weight and tumor diameter were assessed once a week. After receiving medication for nine weeks, every mouse died. The tumor underwent a macroscopical and microcosmic assessment.

### Statistical analysis

All analyses were conducted using the statistical software SPSS22.0 (SPSS Inc., Chicago, IL, USA). The mean ± SD was displayed for the data. One-way analysis and the student’s t-test were employed for comparison. Utilizing the X2 test, classification factors were examined. At *P<0.05*, statistical significance was established.

### Data availability statement

The data that support the findings of this study are available on request from the corresponding author.

## Supplementary Material

Supplementary Table 1
