# Supplementary material for: LncRNA SH3BP5-AS1 promotes hepatocellular carcinoma progression by sponging miR-6838-5p and activation of PTPN4
Source: Aging (Albany NY). 2024 May 16;16(10):8511–23. doi: 10.18632/aging.205811 (PMC11164516; doi:10.18632/aging.205811)
Supplement: Supplementary Table 1 [file aging-16-205811-s001.pdf]

SUPPLEMENTARY TABLE

Supplementary Table 1. The information of primers used in this study.

Gene	Primer	Sequence (5' to 3')
SH3BP5-AS1	Forward	CCGCTCGTGGATCTCATCTG
	Reverse	GCATCTTAAGGCTAGCAGGGT
GAPDH	Forward	GGAGTCCACTGGCGTCTTCA
	Reverse	GTCATGAGTCCTTCCACGATACC
U6	Forward	CTCGCTTCGGCAGCACA
	Reverse	AACGCTTCACGAATTTGCGT
PTPN4	Forward	TCAGAAGATGCCTGTGATTGTGT
	Reverse	TGTCCCGACCATTGATCAGTAC
PDK1	Forward	CTGTGATACGGATCAGAAACCG
	Reverse	TCCACCAAACAATAAAGAGTGCT
PFK1	Forward	GGTGCCCGTGTCTTCTTTGT
	Reverse	AAGCATCATCGAAACGCTCTC
PKM2	Forward	GTCGA AGCCCCATAGTGAAG
	Reverse	GTGAATCAATGTCCAGGCGG
